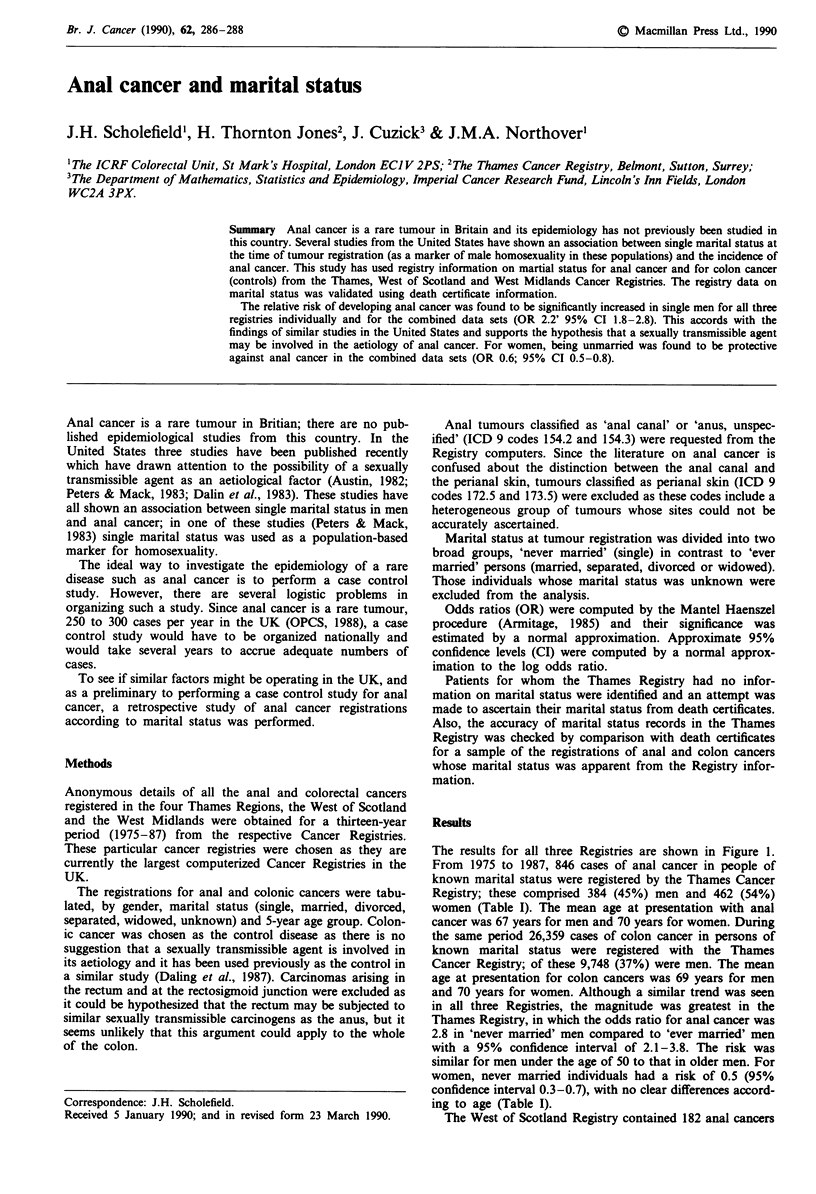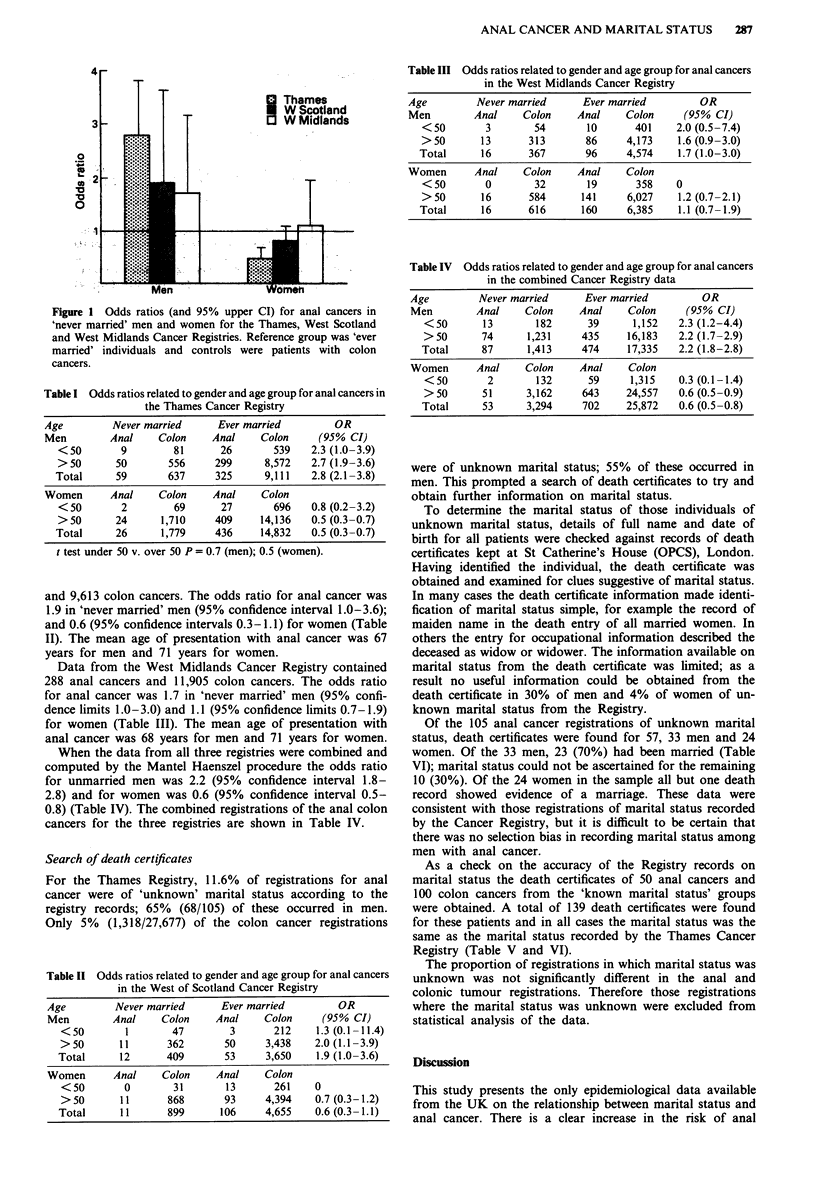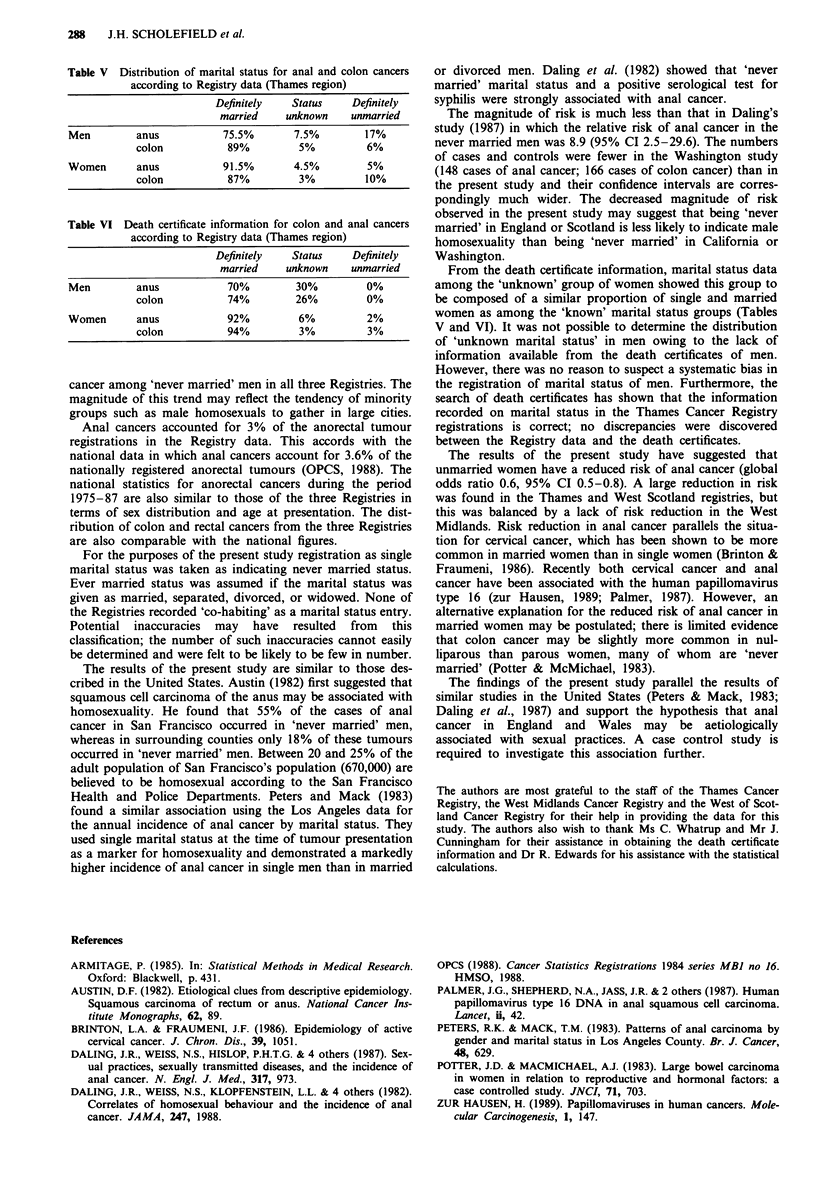# Anal cancer and marital status.

**DOI:** 10.1038/bjc.1990.279

**Published:** 1990-08

**Authors:** J. H. Scholefield, H. Thornton Jones, J. Cuzick, J. M. Northover

**Affiliations:** ICRF Colorectal Unit, St Mark's Hospital, London.

## Abstract

Anal cancer is a rare tumour in Britain and its epidemiology has not previously been studied in this country. Several studies from the United States have shown an association between single marital status at the time of tumour registration (as a marker of male homosexuality in these populations) and the incidence of anal cancer. This study has used registry information on martial status for anal cancer and for colon cancer (controls) from the Thames, West of Scotland and West Midlands Cancer Registries. The registry data on marital status was validated using death certificate information. The relative risk of developing anal cancer was found to be significantly increased in single men for all three registries individually and for the combined data sets (OR 2.2' 95% CI 1.8-2.8). This accords with the findings of similar studies in the United States and supports the hypothesis that a sexually transmissible agent may be involved in the aetiology of anal cancer. For women, being unmarried was found to be protective against anal cancer in the combined data sets (OR 0.6; 95% CI 0.5-0.8).


Br. J. Cancer (1990), 62, 286-288 © Macmillan Press Ltd., 1990

### Anal cancer and marital status

J.H. Scholefield[1], H. Thornton Jones[2], J. Cuzick[3] & J.M.A. Northover[1]

[1]*The ICRF Colorectal Unit, St Mark's Hospital, London EC1V 2PS;* [2]*The Thames Cancer Registry, Belmont, Sutton, Surrey;* [3]*The Department of Mathematics, Statistics and Epidemiology, Imperial Cancer Research Fund, Lincoln's Inn Fields, London WC2A 3PX.*

**Summary** Anal cancer is a rare tumour in Britain and its epidemiology has not previously been studied in this country. Several studies from the United States have shown an association between single marital status at the time of tumour registration (as a marker of male homosexuality in these populations) and the incidence of anal cancer. This study has used registry information on martial status for anal cancer and for colon cancer (controls) from the Thames, West of Scotland and West Midlands Cancer Registries. The registry data on marital status was validated using death certificate information.

The relative risk of developing anal cancer was found to be significantly increased in single men for all three registries individually and for the combined data sets (OR 2.2' 95% CI 1.8-2.8). This accords with the findings of similar studies in the United States and supports the hypothesis that a sexually transmissible agent may be involved in the aetiology of anal cancer. For women, being unmarried was found to be protective against anal cancer in the combined data sets (OR 0.6; 95% CI 0.5-0.8).

Anal cancer is a rare tumour in Britian; there are no published epidemiological studies from this country. In the United States three studies have been published recently which have drawn attention to the possibility of a sexually transmissible agent as an aetiological factor (Austin, 1982; Peters & Mack, 1983; Dalin *et al.*, 1983). These studies have all shown an association between single marital status in men and anal cancer; in one of these studies (Peters & Mack, 1983) single marital status was used as a population-based marker for homosexuality.

The ideal way to investigate the epidemiology of a rare disease such as anal cancer is to perform a case control study. However, there are several logistic problems in organizing such a study. Since anal cancer is a rare tumour, 250 to 300 cases per year in the UK (OPCS, 1988), a case control study would have to be organized nationally and would take several years to accrue adequate numbers of cases.

To see if similar factors might be operating in the UK, and as a preliminary to performing a case control study for anal cancer, a retrospective study of anal cancer registrations according to marital status was performed.

### Methods

Anonymous details of all the anal and colorectal cancers registered in the four Thames Regions, the West of Scotland and the West Midlands were obtained for a thirteen-year period (1975-87) from the respective Cancer Registries. These particular cancer registries were chosen as they are currently the largest computerized Cancer Registries in the UK.

The registrations for anal and colonic cancers were tabulated, by gender, marital status (single, married, divorced, separated, widowed, unknown) and 5-year age group. Colonic cancer was chosen as the control disease as there is no suggestion that a sexually transmissible agent is involved in its aetiology and it has been used previously as the control in a similar study (Daling *et al.*, 1987). Carcinomas arising in the rectum and at the rectosigmoid junction were excluded as it could be hypothesized that the rectum may be subjected to similar sexually transmissible carcinogens as the anus, but it seems unlikely that this argument could apply to the whole of the colon.

Correspondence: J.H. Scholefield.
Received 5 January 1990; and in revised form 23 March 1990.

Anal tumours classified as 'anal canal' or 'anus, unspecified' (ICD 9 codes 154.2 and 154.3) were requested from the Registry computers. Since the literature on anal cancer is confused about the distinction between the anal canal and the perianal skin, tumours classified as perianal skin (ICD 9 codes 172.5 and 173.5) were excluded as these codes include a heterogeneous group of tumours whose sites could not be accurately ascertained.

Marital status at tumour registration was divided into two broad groups, 'never married' (single) in contrast to 'ever married' persons (married, separated, divorced or widowed). Those individuals whose marital status was unknown were excluded from the analysis.

Odds ratios (OR) were computed by the Mantel Haenszel procedure (Armitage, 1985) and their significance was estimated by a normal approximation. Approximate 95% confidence levels (CI) were computed by a normal approximation to the log odds ratio.

Patients for whom the Thames Registry had no information on marital status were identified and an attempt was made to ascertain their marital status from death certificates. Also, the accuracy of marital status records in the Thames Registry was checked by comparison with death certificates for a sample of the registrations of anal and colon cancers whose marital status was apparent from the Registry information.

### Results

The results for all three Registries are shown in Figure 1. From 1975 to 1987, 846 cases of anal cancer in people of known marital status were registered by the Thames Cancer Registry; these comprised 384 (45%) men and 462 (54%) women (Table I). The mean age at presentation with anal cancer was 67 years for men and 70 years for women. During the same period 26,359 cases of colon cancer in persons of known marital status were registered with the Thames Cancer Registry; of these 9,748 (37%) were men. The mean age at presentation for colon cancers was 69 years for men and 70 years for women. Although a similar trend was seen in all three Registries, the magnitude was greatest in the Thames Registry, in which the odds ratio for anal cancer was 2.8 in 'never married' men compared to 'ever married' men with a 95% confidence interval of 2.1-3.8. The risk was similar for men under the age of 50 to that in older men. For women, never married individuals had a risk of 0.5 (95% confidence interval 0.3-0.7), with no clear differences according to age (Table I).

The West of Scotland Registry contained 182 anal cancers

**Figure 1** Odds ratios (and 95% upper CI) for anal cancers in 'never married' men and women for the Thames, West Scotland and West Midlands Cancer Registries. Reference group was 'ever married' individuals and controls were patients with colon cancers.

**Table I** Odds ratios related to gender and age group for anal cancers in the Thames Cancer Registry

| *Age* | *Never married* | | *Ever married* | | *OR* |
|---|---|---|---|---|---|
| Men | *Anal* | *Colon* | *Anal* | *Colon* | *(95% CI)* |
| <50 | 9 | 81 | 26 | 539 | 2.3 (1.0–3.9) |
| >50 | 50 | 556 | 299 | 8,572 | 2.7 (1.9–3.6) |
| Total | 59 | 637 | 325 | 9,111 | 2.8 (2.1–3.8) |
| Women | *Anal* | *Colon* | *Anal* | *Colon* | |
| <50 | 2 | 69 | 27 | 696 | 0.8 (0.2–3.2) |
| >50 | 24 | 1,710 | 409 | 14,136 | 0.5 (0.3–0.7) |
| Total | 26 | 1,779 | 436 | 14,832 | 0.5 (0.3–0.7) |

*t* test under 50 v. over 50 $P = 0.7$ (men); 0.5 (women).

and 9,613 colon cancers. The odds ratio for anal cancer was 1.9 in 'never married' men (95% confidence interval 1.0–3.6); and 0.6 (95% confidence intervals 0.3–1.1) for women (Table II). The mean age of presentation with anal cancer was 67 years for men and 71 years for women.

Data from the West Midlands Cancer Registry contained 288 anal cancers and 11,905 colon cancers. The odds ratio for anal cancer was 1.7 in 'never married' men (95% confidence limits 1.0–3.0) and 1.1 (95% confidence limits 0.7–1.9) for women (Table III). The mean age of presentation with anal cancer was 68 years for men and 71 years for women.

When the data from all three registries were combined and computed by the Mantel Haenszel procedure the odds ratio for unmarried men was 2.2 (95% confidence interval 1.8–2.8) and for women was 0.6 (95% confidence interval 0.5–0.8) (Table IV). The combined registrations of the anal colon cancers for the three registries are shown in Table IV.

### *Search of death certificates*

For the Thames Registry, 11.6% of registrations for anal cancer were of 'unknown' marital status according to the registry records; 65% (68/105) of these occurred in men. Only 5% (1,318/27,677) of the colon cancer registrations

**Table II** Odds ratios related to gender and age group for anal cancers in the West of Scotland Cancer Registry

| *Age* | *Never married* | | *Ever married* | | *OR* |
|---|---|---|---|---|---|
| Men | *Anal* | *Colon* | *Anal* | *Colon* | *(95% CI)* |
| <50 | 1 | 47 | 3 | 212 | 1.3 (0.1–11.4) |
| >50 | 11 | 362 | 50 | 3,438 | 2.0 (1.1–3.9) |
| Total | 12 | 409 | 53 | 3,650 | 1.9 (1.0–3.6) |
| Women | *Anal* | *Colon* | *Anal* | *Colon* | |
| <50 | 0 | 31 | 13 | 261 | 0 |
| >50 | 11 | 868 | 93 | 4,394 | 0.7 (0.3–1.2) |
| Total | 11 | 899 | 106 | 4,655 | 0.6 (0.3–1.1) |

**Table III** Odds ratios related to gender and age group for anal cancers in the West Midlands Cancer Registry

| *Age* | *Never married* | | *Ever married* | | *OR* |
|---|---|---|---|---|---|
| Men | *Anal* | *Colon* | *Anal* | *Colon* | *(95% CI)* |
| <50 | 3 | 54 | 10 | 401 | 2.0 (0.5–7.4) |
| >50 | 13 | 313 | 86 | 4,173 | 1.6 (0.9–3.0) |
| Total | 16 | 367 | 96 | 4,574 | 1.7 (1.0–3.0) |
| Women | *Anal* | *Colon* | *Anal* | *Colon* | |
| <50 | 0 | 32 | 19 | 358 | 0 |
| >50 | 16 | 584 | 141 | 6,027 | 1.2 (0.7–2.1) |
| Total | 16 | 616 | 160 | 6,385 | 1.1 (0.7–1.9) |

**Table IV** Odds ratios related to gender and age group for anal cancers in the combined Cancer Registry data

| *Age* | *Never married* | | *Ever married* | | *OR* |
|---|---|---|---|---|---|
| Men | *Anal* | *Colon* | *Anal* | *Colon* | *(95% CI)* |
| <50 | 13 | 182 | 39 | 1,152 | 2.3 (1.2–4.4) |
| >50 | 74 | 1,231 | 435 | 16,183 | 2.2 (1.7–2.9) |
| Total | 87 | 1,413 | 474 | 17,335 | 2.2 (1.8–2.8) |
| Women | *Anal* | *Colon* | *Anal* | *Colon* | |
| <50 | 2 | 132 | 59 | 1,315 | 0.3 (0.1–1.4) |
| >50 | 51 | 3,162 | 643 | 24,557 | 0.6 (0.5–0.9) |
| Total | 53 | 3,294 | 702 | 25,872 | 0.6 (0.5–0.8) |

were of unknown marital status; 55% of these occurred in men. This prompted a search of death certificates to try and obtain further information on marital status.

To determine the marital status of those individuals of unknown marital status, details of full name and date of birth for all patients were checked against records of death certificates kept at St Catherine's House (OPCS), London. Having identified the individual, the death certificate was obtained and examined for clues suggestive of marital status. In many cases the death certificate information made identification of marital status simple, for example the record of maiden name in the death entry of all married women. In others the entry for occupational information described the deceased as widow or widower. The information available on marital status from the death certificate was limited; as a result no useful information could be obtained from the death certificate in 30% of men and 4% of women of unknown marital status from the Registry.

Of the 105 anal cancer registrations of unknown marital status, death certificates were found for 57, 33 men and 24 women. Of the 33 men, 23 (70%) had been married (Table VI); marital status could not be ascertained for the remaining 10 (30%). Of the 24 women in the sample all but one death record showed evidence of a marriage. These data were consistent with those registrations of marital status recorded by the Cancer Registry, but it is difficult to be certain that there was no selection bias in recording marital status among men with anal cancer.

As a check on the accuracy of the Registry records on marital status the death certificates of 50 anal cancers and 100 colon cancers from the 'known marital status' groups were obtained. A total of 139 death certificates were found for these patients and in all cases the marital status was the same as the marital status recorded by the Thames Cancer Registry (Table V and VI).

The proportion of registrations in which marital status was unknown was not significantly different in the anal and colonic tumour registrations. Therefore those registrations where the marital status was unknown were excluded from statistical analysis of the data.

### Discussion

This study presents the only epidemiological data available from the UK on the relationship between marital status and anal cancer. There is a clear increase in the risk of anal

**Table V** Distribution of marital status for anal and colon cancers according to Registry data (Thames region)

| | | *Definitely married* | *Status unknown* | *Definitely unmarried* |
|---|---|---|---|---|
| Men | anus | 75.5% | 7.5% | 17% |
| | colon | 89% | 5% | 6% |
| Women | anus | 91.5% | 4.5% | 5% |
| | colon | 87% | 3% | 10% |

**Table VI** Death certificate information for colon and anal cancers according to Registry data (Thames region)

| | | *Definitely married* | *Status unknown* | *Definitely unmarried* |
|---|---|---|---|---|
| Men | anus | 70% | 30% | 0% |
| | colon | 74% | 26% | 0% |
| Women | anus | 92% | 6% | 2% |
| | colon | 94% | 3% | 3% |

cancer among 'never married' men in all three Registries. The magnitude of this trend may reflect the tendency of minority groups such as male homosexuals to gather in large cities.

Anal cancers accounted for 3% of the anorectal tumour registrations in the Registry data. This accords with the national data in which anal cancers account for 3.6% of the nationally registered anorectal tumours (OPCS, 1988). The national statistics for anorectal cancers during the period 1975–87 are also similar to those of the three Registries in terms of sex distribution and age at presentation. The distribution of colon and rectal cancers from the three Registries are also comparable with the national figures.

For the purposes of the present study registration as single marital status was taken as indicating never married status. Ever married status was assumed if the marital status was given as married, separated, divorced, or widowed. None of the Registries recorded 'co-habiting' as a marital status entry. Potential inaccuracies may have resulted from this classification; the number of such inaccuracies cannot easily be determined and were felt to be likely to be few in number.

The results of the present study are similar to those described in the United States. Austin (1982) first suggested that squamous cell carcinoma of the anus may be associated with homosexuality. He found that 55% of the cases of anal cancer in San Francisco occurred in 'never married' men, whereas in surrounding counties only 18% of these tumours occurred in 'never married' men. Between 20 and 25% of the adult population of San Francisco's population (670,000) are believed to be homosexual according to the San Francisco Health and Police Departments. Peters and Mack (1983) found a similar association using the Los Angeles data for the annual incidence of anal cancer by marital status. They used single marital status at the time of tumour presentation as a marker for homosexuality and demonstrated a markedly higher incidence of anal cancer in single men than in married or divorced men. Daling *et al.* (1982) showed that 'never married' marital status and a positive serological test for syphilis were strongly associated with anal cancer.

The magnitude of risk is much less than that in Daling's study (1987) in which the relative risk of anal cancer in the never married men was 8.9 (95% CI 2.5–29.6). The numbers of cases and controls were fewer in the Washington study (148 cases of anal cancer; 166 cases of colon cancer) than in the present study and their confidence intervals are correspondingly much wider. The decreased magnitude of risk observed in the present study may suggest that being 'never married' in England or Scotland is less likely to indicate male homosexuality than being 'never married' in California or Washington.

From the death certificate information, marital status data among the 'unknown' group of women showed this group to be composed of a similar proportion of single and married women as among the 'known' marital status groups (Tables V and VI). It was not possible to determine the distribution of 'unknown marital status' in men owing to the lack of information available from the death certificates of men. However, there was no reason to suspect a systematic bias in the registration of marital status of men. Furthermore, the search of death certificates has shown that the information recorded on marital status in the Thames Cancer Registry registrations is correct; no discrepancies were discovered between the Registry data and the death certificates.

The results of the present study have suggested that unmarried women have a reduced risk of anal cancer (global odds ratio 0.6, 95% CI 0.5–0.8). A large reduction in risk was found in the Thames and West Scotland registries, but this was balanced by a lack of risk reduction in the West Midlands. Risk reduction in anal cancer parallels the situation for cervical cancer, which has been shown to be more common in married women than in single women (Brinton & Fraumeni, 1986). Recently both cervical cancer and anal cancer have been associated with the human papillomavirus type 16 (zur Hausen, 1989; Palmer, 1987). However, an alternative explanation for the reduced risk of anal cancer in married women may be postulated; there is limited evidence that colon cancer may be slightly more common in nulliparous than parous women, many of whom are 'never married' (Potter & McMichael, 1983).

The findings of the present study parallel the results of similar studies in the United States (Peters & Mack, 1983; Daling *et al.*, 1987) and support the hypothesis that anal cancer in England and Wales may be aetiologically associated with sexual practices. A case control study is required to investigate this association further.

The authors are most grateful to the staff of the Thames Cancer Registry, the West Midlands Cancer Registry and the West of Scotland Cancer Registry for their help in providing the data for this study. The authors also wish to thank Ms C. Whatrup and Mr J. Cunningham for their assistance in obtaining the death certificate information and Dr R. Edwards for his assistance with the statistical calculations.

### References

ARMITAGE, P. (1985). In: *Statistical Methods in Medical Research*. Oxford: Blackwell, p. 431.

AUSTIN, D.F. (1982). Etiological clues from descriptive epidemiology. Squamous carcinoma of rectum or anus. *National Cancer Institute Monographs*, **62,** 89.

BRINTON, L.A. & FRAUMENI, J.F. (1986). Epidemiology of active cervical cancer. *J. Chron. Dis.*, **39,** 1051.

DALING, J.R., WEISS, N.S., HISLOP, P.H.T.G. & 4 others (1987). Sexual practices, sexually transmitted diseases, and the incidence of anal cancer. *N. Engl. J. Med.*, **317,** 973.

DALING, J.R., WEISS, N.S., KLOPFENSTEIN, L.L. & 4 others (1982). Correlates of homosexual behaviour and the incidence of anal cancer. *JAMA*, **247,** 1988.

OPCS (1988). *Cancer Statistics Registrations* 1984 *series MB1 no 16*. HMSO, 1988.

PALMER, J.G., SHEPHERD, N.A., JASS, J.R. & 2 others (1987). Human papillomavirus type 16 DNA in anal squamous cell carcinoma. *Lancet*, **ii,** 42.

PETERS, R.K. & MACK, T.M. (1983). Patterns of anal carcinoma by gender and marital status in Los Angeles County. *Br. J. Cancer*, **48,** 629.

POTTER, J.D. & MACMICHAEL, A.J. (1983). Large bowel carcinoma in women in relation to reproductive and hormonal factors: a case controlled study. *JNCI*, **71,** 703.

ZUR HAUSEN, H. (1989). Papillomaviruses in human cancers. *Molecular Carcinogenesis*, **1,** 147.